# Systemic metabolic dysfunction is associated with local treatment failure: the role of visceral adiposity index in anti-VEGF resistance in diabetic macular edema

**DOI:** 10.3389/fendo.2026.1801978

**Published:** 2026-03-26

**Authors:** Mi Chen, Ting Jiang, Changbo Hu

**Affiliations:** 1 Department of Ophthalmology, Changde Hospital, Xiangya School of Medicine, Central South University (The First People’s Hospital of Changde City), Changde, Hunan, China; 2 Department of Neurosurgery, Changde Hospital, Xiangya School of Medicine, Central South University (The First People’s Hospital of Changde City), Changde, Hunan, China

**Keywords:** adiposity, anti-VEGF, diabetic, edema, index, macular, resistance, systemic metabolic dysfunction

## Abstract

**Background:**

A substantial number of patients with diabetic macular edema (DME) exhibit resistance to standard anti-vascular endothelial growth factor (anti-VEGF) therapy, representing a major clinical challenge. The Visceral Adiposity Index (VAI), a surrogate for visceral fat dysfunction and systemic metabolic derangement, has been linked to diabetic microvascular complications, but its power to serve as a prognostic marker for local therapeutic failure in DME is unknown. This study aimed to determine if baseline VAI, as a marker of systemic metabolic dysfunction, is associated with resistance to intravitreal anti-VEGF therapy in patients with DME.

**Methods:**

This retrospective cohort study analyzed 298 patients with type 2 diabetes and center-involved DME initiating anti-VEGF therapy between January 2022 and December 2023. VAI was calculated at baseline. The primary outcome was a positive functional response, defined by pre-specified Best-Corrected Visual Acuity (BCVA) improvements at 24 months. Patients not meeting these criteria were classified as exhibiting treatment resistance. Multivariable logistic regression identified independent factors associated with response, ROC curve analysis assessed VAI’s prognostic performance, and Kaplan-Meier analysis evaluated time-to-response.

**Results:**

Patients with treatment resistance (non-responders, 47.0%) had significantly higher baseline VAI scores than responders (4.4 ± 3.0 vs. 2.4 ± 1.9, p<0.001). After adjusting for confounders, a higher VAI was independently associated with a greater likelihood of treatment failure (Adjusted OR for positive response = 0.71 per unit increase; 95% CI: 0.61–0.82; p<0.001). VAI demonstrated acceptable prognostic ability for resistance (AUC = 0.73), with an optimal cut-off of 2.50. Patients with VAI ≥2.50 had a significantly lower and delayed probability of achieving a positive response (log-rank p < 0.0001).

**Conclusion:**

Baseline VAI is a potent, independent prognostic marker associated with local treatment failure in response to anti-VEGF therapy in DME. This study demonstrates that a measure of systemic metabolic dysfunction can correlate with the efficacy of a localized treatment. VAI is an easily calculated, non-invasive index that can effectively risk-stratify patients, identifying those who may require more intensive or alternative management strategies from the outset to overcome this metabolic resistance.

## Introduction

Diabetic macular edema (DME), a sight-threatening consequence of diabetic retinopathy (DR), is the main cause of vision impairment in diabetic patients, significantly contributing to blindness among working-age adults globally (1). It is characterized by the accumulation of fluid in the extracellular space within the macula, leading to distorted or blurred vision (2). The pathophysiology of DME involves a multifactorial interplay including neurodegeneration, vascular dysfunction, and inflammation, driven by chronic hyperglycemia. The current standard of care for center-involving DME is first-line treatment with intravitreal anti-vascular endothelial growth factor (anti-VEGF) agents, such as ranibizumab, bevacizumab, and aflibercept (3). These therapies have well-established efficacy in reducing vascular leakage, alleviating macular edema, and improving visual acuity in a significant proportion of patients (4). However, a substantial subset of patients exhibit resistance or do not respond adequately to this treatment (5).

Retrospective cohort studies and case-control studies have indicated that approximately 40-50% of DME patients may not achieve satisfactory outcomes, defined by insufficient improvement in vision or persistent macular thickening despite ongoing therapy (6). This suboptimal response, or treatment failure, poses a significant clinical challenge, leading to increased treatment burden, higher healthcare costs, and a greater risk of irreversible vision loss (7). Consequently, there is an unmet clinical need to identify reliable prognostic biomarkers that can stratify patients before initiating therapy, enabling a more personalized and proactive management strategy to optimize outcomes (5).

The search for prognostic markers has expanded beyond traditional ocular parameters to encompass systemic factors, recognizing that DME is a local manifestation of systemic metabolic dysregulation. Among these, obesity, particularly visceral adiposity, has emerged as a critical player. Visceral fat is not merely inert tissue but an active endocrine organ that secretes pro-inflammatory cytokines and adipokines, contributing to insulin resistance, chronic low-grade inflammation, and dyslipidemia—all key drivers of diabetic microvascular complications (8). In fact, several studies have established a direct link between visceral obesity, as measured by specific indices, and an increased risk for DR and its progression (9, 10). Within this context, the Visceral Adiposity Index (VAI) has gained attention as a robust, gender-specific marker that integrates anthropometric measures (waist circumference [WC] and body mass index [BMI]) with lipid profiles (triglycerides [TG] and high-density lipoprotein cholesterol [HDL-C]) to reflect visceral fat dysfunction and associated cardiometabolic risk (9, 11). Research has shown that higher VAI values are significantly associated with an increased risk of incident DR and chronic kidney disease, often outperforming conventional measures like BMI (10). For instance, a study using cohort data found that higher VAI was associated with an increased risk of having DR after adjusting for multiple confounders (10). Another study reported that VAI had a prognostic value for chronic kidney disease, with an Area Under the Curve (AUC) of 0.710 in men and 0.772 in women.

Despite the growing evidence linking VAI to the presence and severity of diabetic microvascular complications, its role as a prognostic marker of therapeutic failure remains largely unexplored. While some studies have investigated factors associated with anti-VEGF response in neovascular AMD and DME, focusing on factors like baseline central subfield thickness (CST), early anatomical response, or inflammatory markers from complete blood counts (10, 12), the comprehensive metabolic state captured by VAI has not been systematically evaluated in this specific context. Abdominal obesity itself has been suggested to contribute to “treatment resistance” to anti-VEGF therapy in DME patients (13). Therefore, it is plausible that a more sophisticated measure of visceral adipose tissue function, such as the VAI, could provide superior prognostic information. The hypothesis of this study is that a higher baseline VAI, reflecting a state of severe systemic metabolic dysfunction, is independently associated with local treatment failure in response to intravitreal anti-VEGF therapy in patients with DME.

To our knowledge, this is the first study to investigate the intersection of a comprehensive systemic metabolic index and the efficacy of a localized retinal therapy in DME. By validating VAI as a non-invasive prognostic tool, this research aims to contribute to the development of precision medicine approaches in ophthalmology, allowing clinicians to identify high-risk patients who may require alternative or intensified treatment regimens from the outset of their management. This could ultimately lead to improved visual outcomes and more efficient allocation of healthcare resources for a challenging patient population.

## Methods

### Study design and population

**Figure 1:**
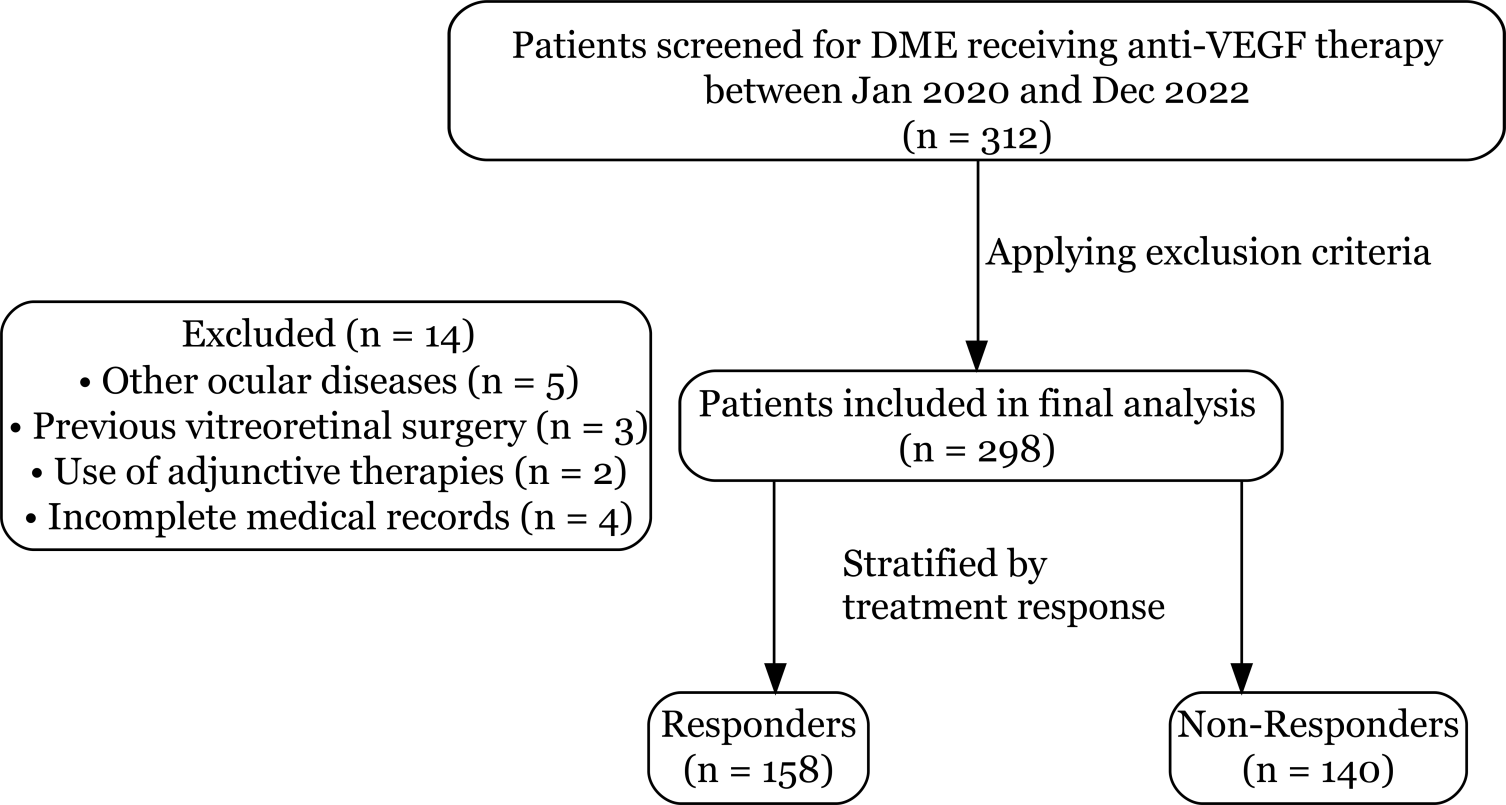
Flow diagram of patient selection. The diagram illustrates the screening, eligibility, and stratification process of the study cohort. A total of 312 patients with Diabetic Macular Edema (DME) were initially screened. After excluding 14 patients based on predefined criteria (other ocular diseases, previous surgery, adjunctive therapies, or incomplete records), 298 patients were included in the final analysis. These patients were then stratified into two groups based on their treatment response: 158 Responders and 140 Non-Responders.

This study was a retrospective cohort analysis conducted at the Department of Ophthalmology, Changde No. 1 People’s Hospital, a tertiary general hospital capable of managing complex ophthalmic conditions. We retrospectively identified and enrolled adult patients diagnosed with center-involved diabetic macular edema who received intravitreal anti-VEGF injections between January 2022 and December 2023. The inclusion criteria were: (1) diagnosis of type 2 diabetes mellitus (T2DM); (2) diagnosis of center-involved DME confirmed by optical coherence tomography (OCT); (3) initiation of intravitreal anti-VEGF therapy; (4) availability of baseline demographic and clinical data required for VAI calculation; and (5) at least two years (24 months) of follow-up data post-treatment initiation. Exclusion criteria included: (1) presence of other significant ocular diseases that could affect vision, such as glaucoma, severe cataract-induced visual impairment, or retinal vein occlusion; (2) history of previous vitreoretinal surgery; (3) use of adjunctive therapies like corticosteroids or laser photocoagulation concurrently with or prior to the initiation of anti-VEGF treatment for DME; and (4) incomplete medical records preventing the calculation of VAI or assessment of treatment response. A patient flow diagram was constructed following the STROBE guidelines to transparently report the process of participant selection (**Figure 1**).

### Outcome definitions

The primary outcome of this study was a positive functional response to anti-VEGF therapy, defined as a meaningful improvement in Best-Corrected Visual Acuity (BCVA). To ensure clinical relevance, we adopted a dynamic threshold definition based on the initial level of vision, as proposed by the Diabetic Retinopathy Clinical Research (DRCR) Network (14). Specifically, a positive VA response was defined as follows: a gain of at least 15 ETDRS letters if the baseline BCVA is equivalent to 20/80 to 20/320 Snellen; a gain of at least 10 ETDRS letters if the baseline BCVA is equivalent to 20/40 to 20/63 Snellen; or a gain of at least 5 ETDRS letters if the baseline BCVA is equivalent to 20/25 to 20/32 Snellen. Patients who met these thresholds at the 24-month follow-up were classified as “responders,” while those who did not were classified as “non-responders,” representing the treatment failure or resistance group. This approach accounts for the principle that a fixed number of letters gained has different clinical significance depending on the starting point of vision (14).

### Data collection and variables

All relevant clinical data were extracted from the electronic medical records of Changde No. 1 People’s Hospital. Baseline demographic and clinical characteristics were collected for all participants. Key variables included age, sex, duration of diabetes, HbA1c levels, and medication history. Anthropometric measurements, including waist circumference (WC) in centimeters and Body Mass Index (BMI) in kg/m², were recorded. Laboratory values for fasting triglycerides (TG) and high-density lipoprotein cholesterol (HDL-C), all in mmol/L, were obtained from routine blood tests conducted close to the baseline visit. BCVA was recorded in Early Treatment Diabetic Retinopathy Study (ETDRS) letters and subsequently converted to logMAR for multivariable modeling. Central retinal thickness (CRT) was measured via OCT at baseline and during follow-up visits. The Visceral Adiposity Index (VAI) was calculated for each patient using gender-specific formulas validated in large cohorts (15, 16). The formula for males is: VAI = [WC/(39.68 + (1.88 × BMI))] × [(TG/1.03) × (1.31/HDL-C)]. For females, the formula is: VAI = [WC/(36.58 + (1.89 × BMI))] × [(TG/0.81) × (1.51/HDL-C)]. As a surrogate for systemic metabolic dysfunction, VAI provides a comprehensive, non-invasive assessment. As the study was conducted in China, we also calculated the Chinese Visceral Adiposity Index (CVAI) for a planned sensitivity analysis, which incorporates age as an additional variable and may offer better prediction for the local population (17, 18).

### Statistical analysis

Statistical analyses were performed using R software (Version 4.1.2; R Foundation for Statistical Computing, Vienna, Austria). Continuous variables were presented as mean ± standard deviation (normally distributed) or median (interquartile range) (skewed distribution), while categorical variables were presented as frequencies and percentages. Between-group comparisons were made using Student’s t-test or Mann-Whitney U test for continuous variables, and the chi-square test or Fisher’s exact test for categorical variables. To assess the association between baseline VAI and treatment response, a multivariable logistic regression model was constructed to evaluate whether VAI was an independent factor associated with a positive treatment response after adjusting for potential confounding variables, including age, sex, diabetes duration, HbA1c, baseline CRT, baseline BCVA (logMAR), and hypertension status. To prevent multicollinearity, individual components of the VAI (BMI, waist circumference, triglycerides, and HDL-C) were strictly excluded from the multivariable models evaluating VAI. Variance Inflation Factors (VIF) were calculated to confirm the absence of significant multicollinearity. Model calibration was assessed using the Hosmer-Lemeshow goodness-of-fit test. Furthermore, VAI was evaluated in quartiles to improve clinical interpretability. For sensitivity analysis, both VAI and CVAI were standardized (per 1 Standard Deviation increase) to allow for a direct comparison of their effect sizes. The results were expressed as adjusted odds ratios (AORs) with 95% confidence intervals (CIs). Receiver Operating Characteristic (ROC) curve analysis was used to evaluate the diagnostic accuracy of VAI in identifying treatment failure. The area under the ROC curve (AUC) was calculated. The optimal cut-off value for VAI was determined using the Youden index. Furthermore, Kaplan-Meier curves were generated to compare the time to achieving a positive treatment response between patients in the high-VAI and low-VAI groups, with differences assessed using the log-rank test. All statistical tests were two-sided, and a p-value of less than 0.05 was considered statistically significant.

### Ethical considerations

This retrospective study protocol was reviewed and approved by the Ethics Committee of Changde No. 1 People’s Hospital. Given the retrospective nature of the study and the use of anonymized data, the requirement for informed consent from individual patients was waived. All data were handled confidentially, with personal identifiers removed to protect patient privacy, in accordance with the principles outlined in the Declaration of Helsinki.

## Results

### Patient characteristics

A total of 312 patients with center-involved diabetic macular edema who received anti-VEGF therapy were initially screened from the hospital database. After applying the exclusion criteria—which included other ocular diseases (n=5), previous vitreoretinal surgery (n=3), use of adjunctive therapies (n=2), and incomplete medical records (n=4)—a final cohort of 298 patients was included in the analysis. The patient selection process is summarized in the flow diagram (**Figure 1**). Of these, 158 patients (53.0%) were classified as “responders” based on the predefined improvement in best-corrected visual acuity (BCVA), while 140 patients (47.0%) were classified as “non-responders,” representing the treatment resistance group.

The baseline demographic and clinical characteristics of the two groups are presented in **Table 1**. There were no significant differences in age or sex between responders and non-responders. However, several metabolic and clinical parameters showed significant inter-group differences. In line with the study hypothesis, non-responders exhibited a markedly worse metabolic profile compared to responders. Specifically, non-responders had a significantly higher baseline VAI score (4.4 ± 3.0 vs. 2.4 ± 1.9, p<0.001), longer duration of diabetes (14.4 ± 6.4 vs. 11.7 ± 6.6 years, p<0.001), higher glycated hemoglobin (HbA1c) (8.3 ± 1.1% vs. 7.8 ± 1.3%, p<0.001), and a higher prevalence of hypertension (73% vs. 52%, p<0.001). Non-responders also presented with a more severe disease state anatomically, with higher mean baseline central retinal thickness (CRT) (499.1 ± 91.9 μm vs. 424.7 ± 92.4 μm, p<0.001). The components of VAI were also significantly worse in the non-responder group, including higher waist circumference (96.7 ± 9.1 cm vs. 90.8 ± 9.1 cm, p<0.001), Body Mass Index (26.8 ± 3.4 kg/m² vs. 26.0 ± 3.8 kg/m², p=0.046), and triglyceride levels (2.7 ± 1.0 mmol/L vs. 1.9 ± 0.9 mmol/L, p<0.001), along with lower high-density lipoprotein cholesterol (HDL-C) levels (1.0 ± 0.3 mmol/L vs. 1.2 ± 0.3 mmol/L, p<0.001).

**Table 1:** Baseline characteristics of study population.

Characteristic	Responders (n=158)	Non-Responders (n=140)	P value
Age, years	64.9 ± 8.2	63.0 ± 8.7	0.098
Male Sex, n (%)	79 (56%)	84 (53%)	0.572
Diabetes Duration, years	11.7 ± 6.6	14.4 ± 6.4	<0.001
Waist Circumference, cm	90.8 ± 9.1	96.7 ± 9.1	<0.001
Body Mass Index, kg/m²	26.0 ± 3.8	26.8 ± 3.4	0.046
Glycated Hemoglobin (HbA1c), %	7.8 ± 1.3	8.3 ± 1.1	<0.001
Triglycerides, mmol/L	1.9 ± 0.9	2.7 ± 1.0	<0.001
High-Density Lipoprotein Cholesterol, mmol/L	1.2 ± 0.3	1.0 ± 0.3	<0.001
Hypertension, n (%)	82 (52%)	102 (73%)	<0.001
Baseline Central Retinal Thickness, μm	424.7 ± 92.4	499.1 ± 91.9	<0.001
Baseline Best-Corrected Visual Acuity, ETDRS letters	64.7 ± 12.2	58.6 ± 13.6	<0.001
Visceral Adiposity Index	2.4 ± 1.9	4.4 ± 3.0	<0.001
Chinese Visceral Adiposity Index	124.8 ± 40.9	153.8 ± 38.0	<0.001

Data are presented as mean ± standard deviation or n (%). P-values are derived from Student’s t-test for continuous variables and chi-square test for categorical variables, comparing Responders and Non-Responders. P-values are rounded to three decimal places; values <0.001 are shown as <0.001.

### VAI as an independent correlate of treatment response

**Figure 2:**
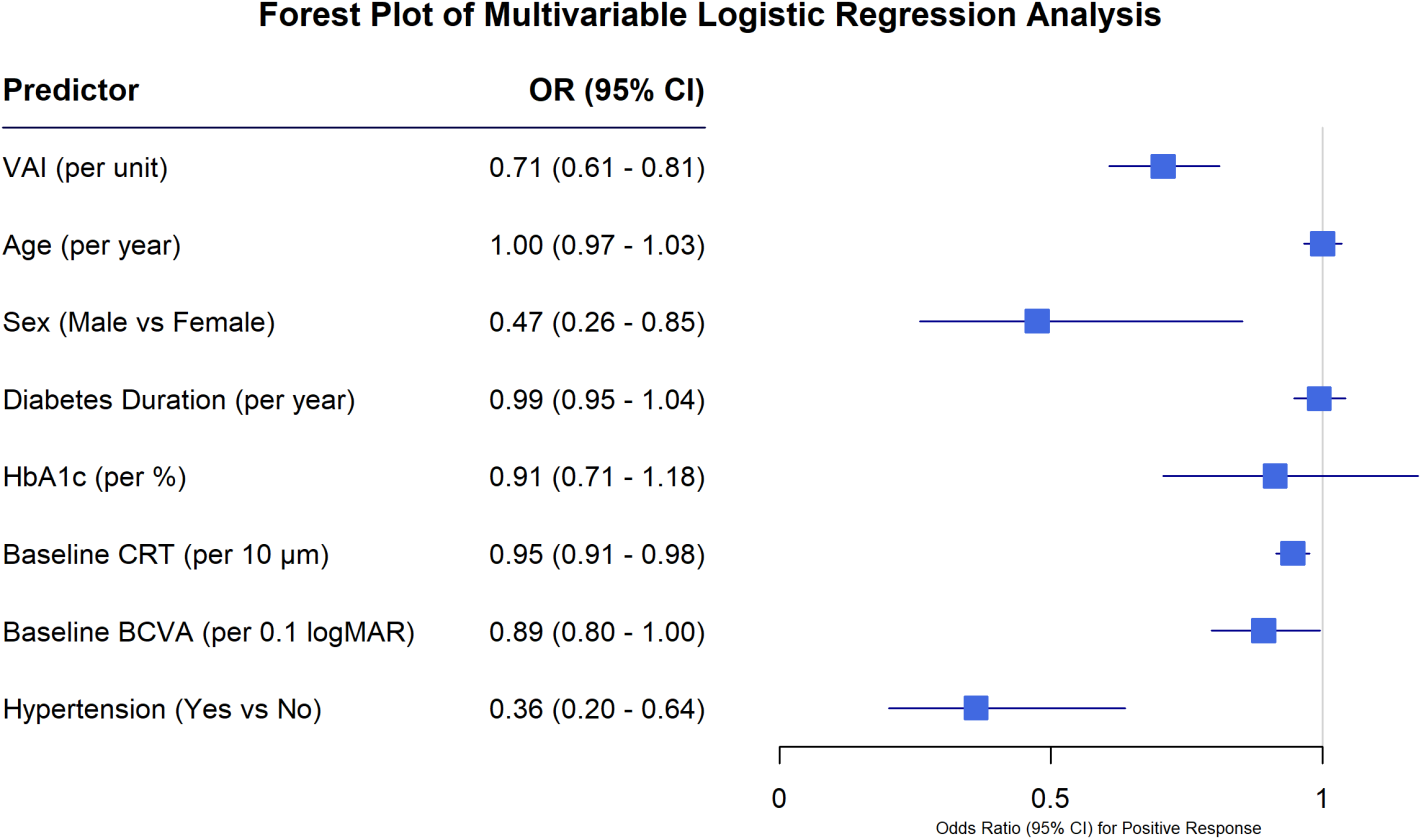
Forest plot of multivariable logistic regression analysis for positive response. The plot graphically presents the adjusted odds ratios (ORs) and 95% confidence intervals (CIs) for factors associated with a positive treatment response from the fully adjusted model. Squares represent the point estimate of the OR, and the horizontal lines extending from the squares represent the 95% CI. The vertical line at an OR of 1.0 indicates no effect. Variables with CIs entirely to the left of the line (VAI, Sex [Male vs Female], Baseline CRT [per 10 μm], Baseline BCVA, and Hypertension) are significantly associated with a lower odds of achieving a positive response to anti-VEGF therapy, indicating they are risk factors for treatment failure.

To identify independent factors associated with a positive treatment response, a multivariable logistic regression analysis was performed. The results are summarized in the forest plot (**Figure 2**) and detailed in **Table 2**. After adjusting for potential confounding factors, including age, sex, diabetes duration, HbA1c, baseline CRT, baseline BCVA (logMAR), and hypertension status, VAI remained a significant independent prognostic marker of treatment outcome. The fully adjusted model showed that for each one-unit increase in VAI, the odds of achieving a positive response decreased by 29% (Adjusted OR (AOR) = 0.71, 95% CI: 0.61–0.82, p<0.001), meaning a higher VAI strongly correlates with treatment failure. Furthermore, multicollinearity diagnostics confirmed that all variables in the fully adjusted model had a VIF well below the threshold of 5 (range: 1.031 to 1.332), validating the stability of the regression estimates (**Supplementary Figure 1**). The Hosmer-Lemeshow goodness-of-fit test for Model 3 indicated adequate calibration (p = 0.137) (**Supplementary Figure 2**).

**Table 2:** Univariate and multivariable logistic regression analysis for evaluating factors associated with treatment response.

Variable	Model 1 (Crude)	Model 2 (Adjusted for Age, Sex)	Model 3 (Fully adjusted)
Visceral Adiposity Index (per unit)	0.70 (0.62-0.79) *	0.66 (0.58-0.76) *	0.71 (0.61-0.82) *
Age (per year)	0.97 (0.95-1.00)	0.99 (0.96-1.02)	1.00 (0.97-1.03)
Male Sex	0.88 (0.55-1.38)	0.46 (0.27-0.79) *	0.47 (0.26-0.86) *
Diabetes Duration (per year)	0.94 (0.90-0.97) *	0.94 (0.91-0.98) *	0.99 (0.95-1.04)
HbA1c (per %)	0.67 (0.55-0.82) *	0.68 (0.56-0.83) *	0.91 (0.71-1.18)
Baseline CRT (per 10 μm)	0.92 (0.89-0.95)*	0.92 (0.90-0.95) *	0.95 (0.91-0.98) *
Baseline BCVA (per 0.1 logMAR increase)	0.83 (0.76-0.91) *	0.84 (0.76-0.92) *	0.89 (0.80-1.00) *
Hypertension	0.40 (0.25-0.65) *	0.40 (0.24-0.65) *	0.36 (0.20-0.64) *
Constant	3.49	11.43	545.81

The table shows Odds Ratios (ORs) and 95% Confidence Intervals (CIs) for identifying a positive treatment response. *Indicates statistical significance (p<0.05). Model 1: Crude univariate model for each predictor. Model 2: Adjusted for age and sex. Model 3: Fully adjusted model including VAI, age, sex, diabetes duration, HbA1c, baseline CRT, baseline BCVA, and hypertension. CRT, central retinal thickness. The Hosmer-Lemeshow goodness-of-fit test for Model 3 indicated adequate calibration (p = 0.137).

To improve clinical interpretability, VAI was also analyzed in quartiles. Patients in the highest VAI quartile (Q4) demonstrated significantly lower odds of achieving a positive treatment response compared to those in the lowest quartile (Q1) (AOR 0.08, 95% CI 0.03-0.22, p < 0.001) (**Supplementary Figure 3**). Other significant independent factors associated with a poorer response included male sex (AOR = 0.47, 95% CI: 0.26–0.86), higher baseline CRT (AOR per 10 μm = 0.95, 95% CI: 0.91–0.98), worse baseline BCVA (AOR per 0.1 logMAR increase = 0.89, 95% CI: 0.80–1.00), and the presence of hypertension (AOR = 0.36, 95% CI: 0.20–0.64).

### Diagnostic and prognostic performance of VAI

**Figure 3:**
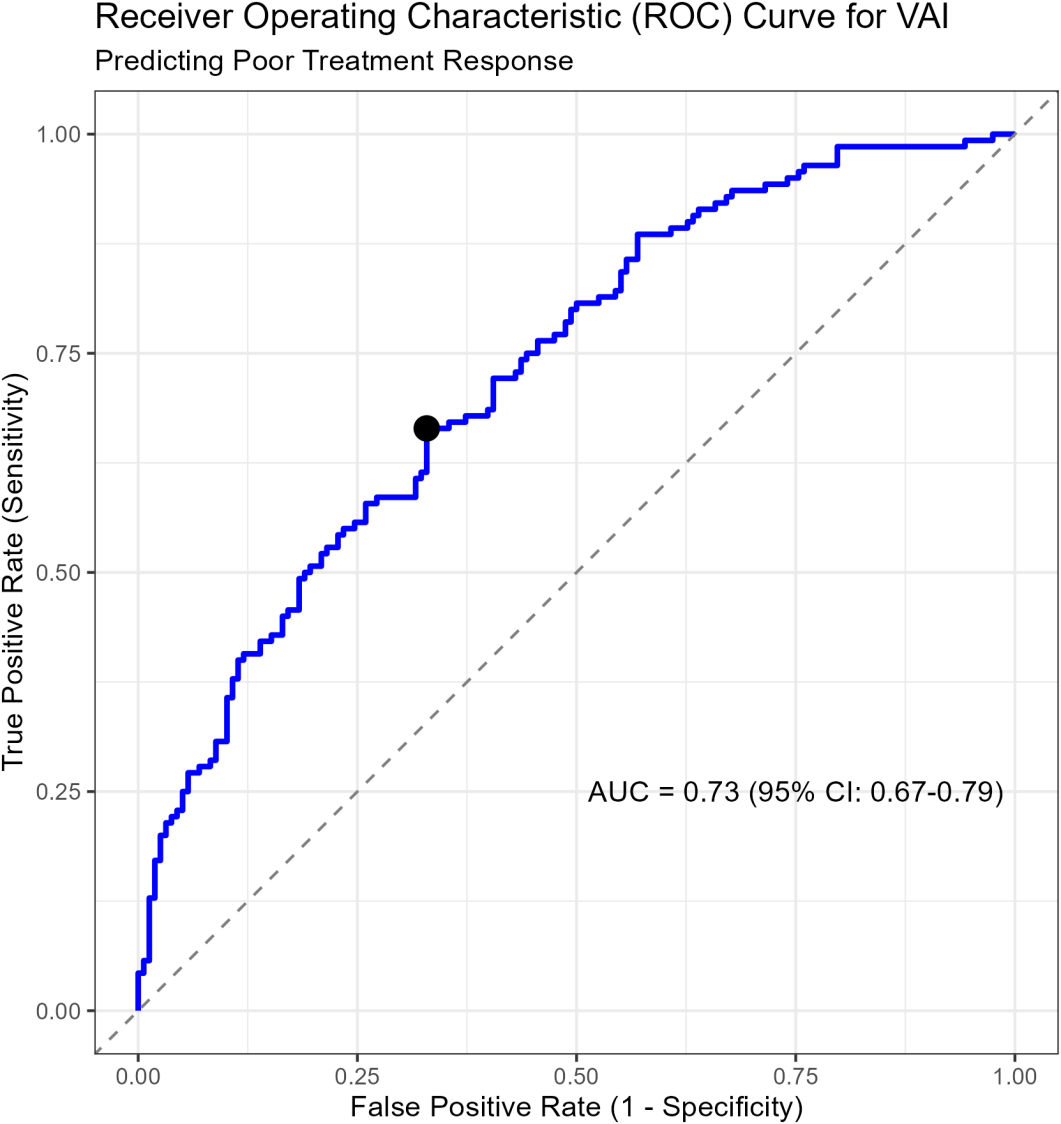
Receiver operating characteristic (ROC) curve for VAI in identifying treatment resistance. The curve plots the true positive rate (sensitivity) against the false positive rate (1-specificity) for various cut-off points of the Visceral Adiposity Index (VAI) in identifying treatment resistance (non-response). The Area Under the Curve (AUC) is 0.73 (95% CI: 0.67–0.79), indicating acceptable prognostic performance. The diagonal dashed line represents a test with no discriminatory ability (AUC = 0.5).

The diagnostic performance of baseline VAI in identifying treatment resistance was evaluated using ROC curve analysis (**Figure 3**). The VAI demonstrated acceptable prognostic ability, with an Area Under the Curve (AUC) of 0.73 (95% CI: 0.67–0.79). The optimal cut-off value for VAI, determined by the Youden index, was 2.50. At this threshold, VAI showed a sensitivity of 66.4% and a specificity of 67.1% for identifying non-response (**Table 3**). These findings suggest that VAI provides clinically useful information for risk stratification. A sensitivity analysis comparing standardized VAI with the Chinese Visceral Adiposity Index (CVAI) confirmed that both were significant correlates (VAI adjusted OR per 1 SD increase: 0.40; CVAI adjusted OR per 1 SD increase: 0.44), although VAI demonstrated slightly better prognostic performance (AUC 0.73 vs 0.69) (**Supplementary Figure 4**).

**Table 3:** Diagnostic performance of VAI in identifying poor treatment response.

Metric	Value
Area Under the Curve (95% CI)	0.73 (0.67 - 0.79)
Optimal Cut-off Value	2.50
Sensitivity	66.4%
Specificity	67.1%
Positive Likelihood Ratio (+LR)	2.02
Negative Likelihood Ratio (-LR)	0.50
Youden Index	1.34

The table summarizes the diagnostic performance of the Visceral Adiposity Index (VAI) in identifying treatment failure (non-response). CI = Confidence Interval.

**Figure 4:**
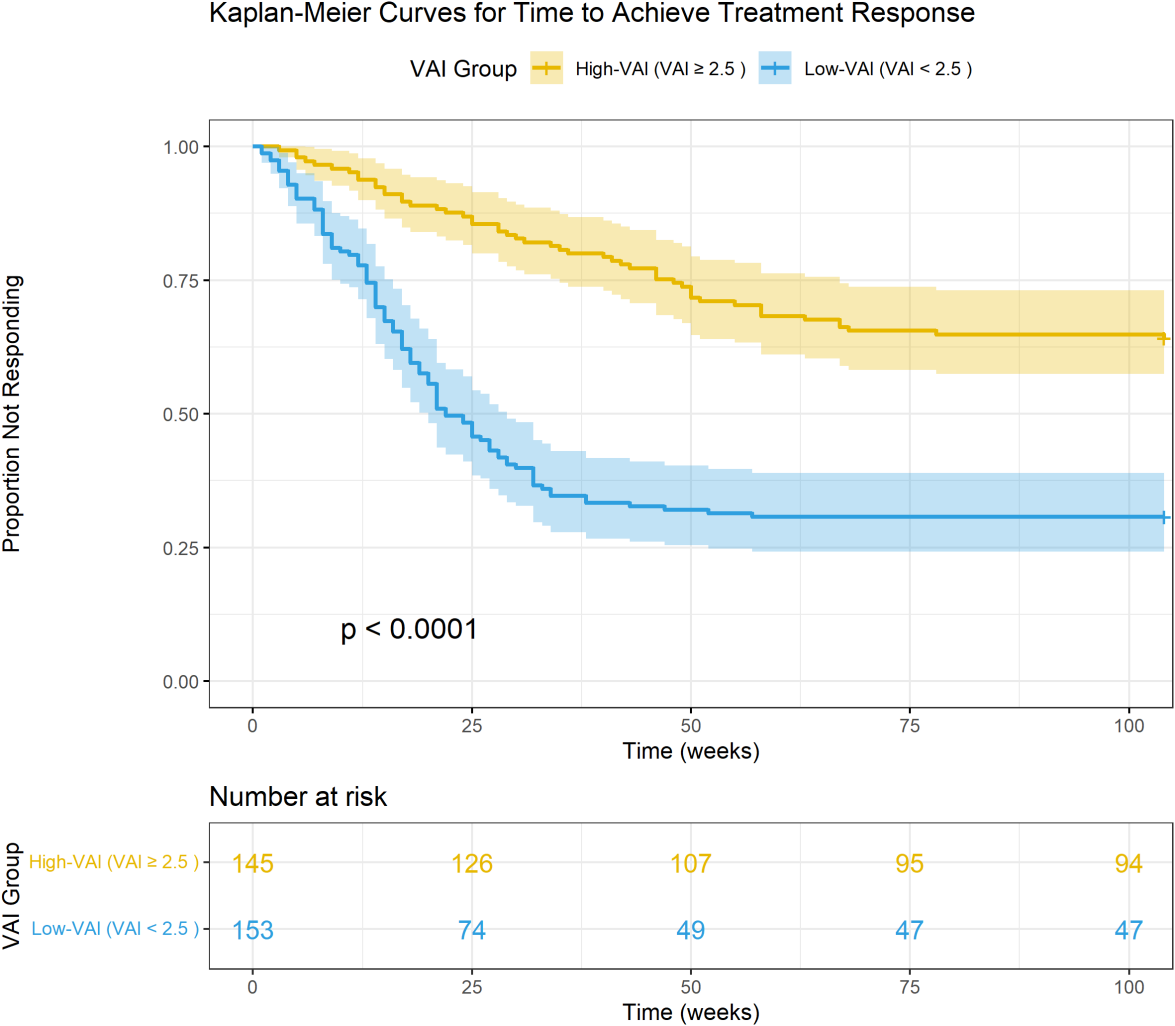
Kaplan-Meier curves for time to achieve treatment response. The figure displays the proportion of patients who have achieved a positive treatment response over 100 weeks, stratified by Visceral Adiposity Index (VAI) group. The cohort was dichotomized at the optimal VAI cut-off of 2.50, identified from ROC analysis. The High-VAI group (VAI ≥ 2.50) demonstrated a significantly lower cumulative probability of achieving a response compared to the Low-VAI group (VAI < 2.50). The log-rank test showed a highly significant difference between the groups (p < 0.0001).

To assess the prognostic value of VAI over time, Kaplan-Meier analysis was used to compare the time to achieving a positive treatment response between the high-VAI group (VAI ≥ 2.50) and the low-VAI group (VAI < 2.50). The cumulative probability of achieving a positive response was significantly lower in the high-VAI group compared to the low-VAI group (**Figure 4**). The median time to response was prolonged in the high-VAI group, and the difference between the two curves was highly statistically significant (Log-rank P < 0.0001). This indicates that patients with greater systemic metabolic dysfunction, as captured by VAI, not only have a lower overall probability of responding but also take longer to reach a favorable outcome if they respond at all.

### Correlation of VAI with other metabolic variables

**Figure 5:**
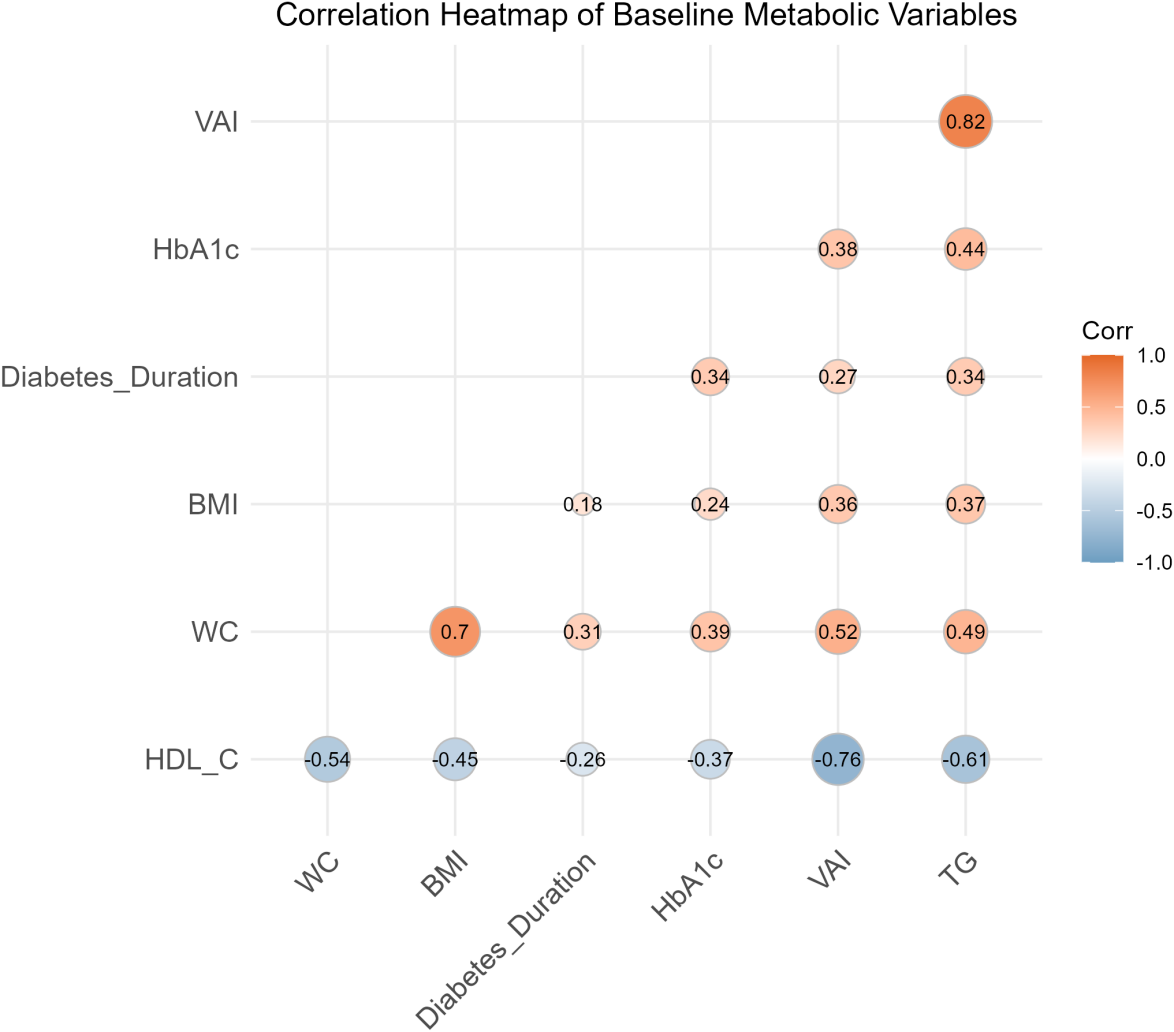
Correlation heatmap of baseline metabolic variables. This heatmap visualizes the Pearson correlation coefficients (r) between the Visceral Adiposity Index (VAI) and other key baseline metabolic and clinical variables. The color scale indicates the direction and strength of the correlation, with orange/red representing positive correlations and blue representing negative correlations. VAI shows a strong positive correlation with triglycerides (TG; r=0.82), a moderate positive correlation with waist circumference (WC; r=0.52), and a strong negative correlation with HDL-cholesterol (HDL_C; r=-0.76). It also shows a moderate positive correlation with HbA1c (r=0.49) and weaker positive correlations with diabetes duration (r=0.39) and BMI (r=0.31). This highlights VAI’s role as a composite index of systemic metabolic health.

A correlation heatmap was generated to visualize the relationships between VAI and other baseline metabolic and clinical variables (**Figure 5**). VAI showed a strong positive correlation with triglycerides (r=0.82, p<0.001) and a strong negative correlation with HDL-C (r=−0.76, p<0.001), underscoring its robust integration of lipid parameters. It also demonstrated moderate positive correlations with waist circumference (r=0.52, p<0.001) and a weak positive correlation with BMI (r=0.31, p<0.001). Furthermore, VAI was moderately correlated with HbA1c (r=0.49, p<0.001) and weakly with the duration of diabetes (r=0.39, p<0.001), reinforcing its role as a comprehensive marker linking visceral adiposity to overall systemic metabolic dysfunction.

## Discussion

This retrospective cohort study provides compelling evidence that a higher baseline Visceral Adiposity Index (VAI) is an independent prognostic marker associated with a local treatment failure to intravitreal anti-VEGF therapy in patients with diabetic macular edema (DME). Our analysis of 298 patients revealed that for every one-unit increase in VAI, the likelihood of a positive visual outcome decreases by 29%, even after rigorous adjustment for a comprehensive set of demographics, metabolic, and ocular confounders (AOR 0.71, 95% CI 0.61-0.82). Furthermore, patients with a VAI at or above the identified optimal cut-off of 2.50 not only had a significantly lower cumulative probability of achieving a positive response but also experienced a markedly delayed time to that response. These findings, supported by a sensitivity analysis using the Chinese Visceral Adiposity Index (CVAI), establish VAI as a potent, non-invasive biomarker for identifying resistance to localized therapy based on systemic metabolic status, bridging the gap between systemic health and local therapeutic efficacy.

The biological plausibility of our findings is deeply rooted in the pathophysiology of DME, which is increasingly understood as a local manifestation of systemic metabolic derangement. VAI is not merely an indicator of fat quantity but a validated surrogate for visceral adipocyte dysfunction, integrating both anthropometric (waist circumference, BMI) and lipid (triglycerides, HDL-C) parameters (9, 19). Visceral adipose tissue is a highly active endocrine and metabolic organ that, in a state of dysfunction, orchestrates a milieu of chronic low-grade inflammation, profound insulin resistance, and atherogenic dyslipidemia (20, 21). Rather than just a storage site, inflamed visceral fat acts as a “reservoir” for chronic systemic inflammation, establishing a systemic-to-local inflammatory axis that directly impacts retinal microvascular environments. The significance of VAT metabolic activity in retinal pathologies has been recently established through objective measurements using 18F-FDG PET/CT imaging. For instance, increased metabolic activity in visceral fat is significantly associated with age-related macular degeneration (AMD) (22, 23), reinforcing the functional link between active visceral fat deposits and ocular disease. This toxic systemic environment directly impacts the retinal microvasculature. Pro-inflammatory cytokines such as TNF-α and IL-6, released by hypertrophic adipocytes and macrophages, enhance the expression of adhesion molecules on retinal endothelial cells, promoting leukostasis and leading to the breakdown of the blood-retinal barrier (BRB) (8, 24–26). Concurrently, the dyslipidemia captured by VAI—specifically elevated triglycerides and reduced HDL-C, as strongly correlated in our data (r=0.82 and r=-0.76, respectively)—exacerbates endothelial dysfunction and oxidative stress (11). While anti-VEGF agents are highly effective in neutralizing VEGF, their primary target, they may be insufficient to overcome the multifactorial assault on the BRB sustained by this underlying metabolic turmoil. The persistent systemic pro-inflammatory and pro-angiogenic state driven by dysfunctional visceral adipose tissue likely renders the local retinal microenvironment resistant to a VEGF-specific blockade, creating a level of pathophysiological pressure that exceeds the therapeutic capacity of VEGF neutralization alone (10, 11, 27). The systemic inflammatory burden and metabolic stress originating from this active visceral fat are likely key drivers of the chronic inflammation and suboptimal treatment response observed in our cohort. This hypothesis is strongly supported by our baseline data, where the non-responder group exhibited a significantly more hostile metabolic profile across every measured component of the VAI and CVAI, in addition to poorer glycemic control and longer diabetes duration.

Our results align with and substantively extend the existing literature. Numerous longitudinal studies have previously established a robust association between higher VAI and an increased risk for the incidence and progression of diabetic retinopathy (DR) (9, 10, 28). For instance, a large prospective cohort study demonstrated that elevated VAI levels were a significant factor associated with the development of DR over a multi-year follow-up period (10). Our study advances this paradigm by being one of the first to pivot from disease risk to therapeutic failure, demonstrating that the same metabolic index that correlates with the onset of retinopathy also correlates with its resistance to standard-of-care treatment. This finding bridges the critical gap between understanding the pathogenesis of diabetic complications and navigating the practical challenges of their management. Furthermore, our multivariable model confirmed the role of other known risk factors, reinforcing the validity of our analysis. The strong association between higher baseline CRT and poor response (AOR 0.95 per 10 μm) is consistent with previous reports suggesting that more severe anatomical disruption at onset portends a more challenging treatment course. Similarly, the link between hypertension and refractory DME (AOR 0.36) is well-established, as elevated systemic blood pressure exacerbates hydrostatic pressure within the retinal capillaries, promoting vascular leakage (29–31). The finding that male sex was an independent correlate of poorer response (AOR 0.47) is a noteworthy observation in this specific context. While the underlying mechanisms are not fully elucidated, this may reflect unmeasured confounding from lifestyle factors, hormonal differences influencing inflammatory pathways, or variations in healthcare-seeking behavior, and clearly warrants further investigation.

The primary advantage of VAI, and a key strength of this study, lies in its remarkable simplicity and accessibility. Unlike sophisticated imaging techniques for quantifying visceral fat, VAI is calculated from four routine, low-cost measurements, making it immediately applicable in virtually any clinical setting worldwide. The clinical implications are therefore profound and immediate. Our ROC analysis, which yielded an AUC of 0.73, indicates acceptable discriminatory power, outperforming many single biomarkers and positioning VAI as a clinically useful risk-stratification tool. The identification of a VAI cut-off of 2.50, which demonstrated a sensitivity of 66.4% and specificity of 67.1% for detecting non-responders, provides clinicians with a practical threshold. A baseline VAI exceeding this value should act as a “red flag,” signaling a high risk of metabolic resistance to therapy and prompting a more proactive and personalized treatment strategy from the outset. As powerfully illustrated by our Kaplan-Meier analysis, these high-VAI patients are not only less likely to respond but also respond much more slowly. Delaying a more aggressive intervention in this group likely condemns them to prolonged periods of suboptimal vision and an increased risk of irreversible photoreceptor damage. For these high-risk individuals, clinicians might consider initiating therapy with a more potent anti-VEGF agent, employing a treat-and-extend regimen with shorter initial intervals to gain rapid control of the edema, or even contemplating early combination therapy with intravitreal corticosteroids to more directly address the heightened inflammatory component of their disease. Implementing such a VAI-guided, proactive strategy has the potential to break the cycle of treatment failure, improve long-term visual outcomes, and ultimately reduce the cumulative treatment burden on both patients and healthcare systems.

Despite its significant findings, this study is not without limitations, primarily those inherent to its retrospective, single-center design. Although we used multivariable regression to control for a wide array of potential confounders, the possibility of selection bias or residual confounding from unmeasured variables (e.g., specific anti-VEGF agent used, dosing frequency, detailed dietary and exercise habits, or genetic predispositions) cannot be entirely eliminated. Second, we evaluated model calibration using the Hosmer-Lemeshow goodness-of-fit test, which indicated adequate fit (p = 0.137). However, we did not perform internal validation such as bootstrapping. Third, causal inference is limited due to the observational design; therefore, VAI should be viewed as a marker of association rather than a direct causative factor. Finally, the optimal VAI cut-off of 2.50 was derived from our specific ethnically homogeneous Chinese cohort. Body fat distribution and metabolic profiles vary significantly across races; thus, this specific cut-off requires external validation and may not be generalizable to non-Chinese populations. Additionally, while a sensitivity analysis using the Chinese Visceral Adiposity Index (CVAI) was performed, which also showed a significant association with treatment response, its prognostic utility was modestly lower than the standard VAI in our cohort. This suggests that while ethnicity-specific indices are valuable, the standard VAI remains a robust prognostic marker in this context, though further comparative studies are warranted.

## Conclusion

This study concludes that a higher baseline Visceral Adiposity Index (VAI) is a powerful, independent prognostic marker associated with local treatment failure in response to anti-VEGF therapy in patients with diabetic macular edema. As a key indicator of systemic metabolic dysfunction, an elevated VAI is associated with significantly lower rates of visual improvement and delayed treatment efficacy. As a simple, non-invasive tool derived from routine measurements, VAI offers immense clinical utility for risk stratification. Its integration into practice can help identify high-risk patients early, enabling the timely implementation of personalized and more aggressive treatment strategies. This approach, which links systemic health to local ocular outcomes, is pivotal for advancing precision management in diabetic eye disease.

## Data Availability

The raw data supporting the conclusions of this article will be made available by the authors, without undue reservation.

## References

[1] Zhang J Zhang J Zhang C Zhang J Gu L Luo D . Diabetic macular edema: current understanding, molecular mechanisms and therapeutic implications. Cells. (2022) 11(21). doi: 10.3390/cells11213362. PMID: 36359761 PMC9655436

[2] Prince J Kumar D Ghosh A Arevalo JF Zhang AY . Surgical management of diabetic macular edema. Curr Diabetes Rep. (2023) 23:119–25. doi: 10.1007/s11892-023-01505-3. PMID: 37043090

[3] Shimura M Hirano T Tsuiki E Takamura Y Morizane Y Akiyama K . Alteration of treatment choices and the visual prognosis for diabetic macular edema in the era of anti-vascular endothelial growth factor drugs: analysis of the STREAT-DME 2 study. Retina (Philadelphia Pa). (2025) 45:335–44. doi: 10.1097/IAE.0000000000004301. PMID: 39423137 PMC11753449

[4] Sadiq MA Halim MS Hassan M Onghanseng N Karaca I Agarwal A . Pharmacological agents in development for diabetic macular edema. Int J Retina Vitreous. (2020) 6:29. doi: 10.1186/s40942-020-00234-z. PMID: 32670612 PMC7341631

[5] Mondal A Nandi A Pramanik S Mondal LK . Application of deep learning algorithm for judicious use of anti-VEGF in diabetic macular edema. Sci Rep. (2025) 15:4569. doi: 10.1038/s41598-025-87290-3. PMID: 39915516 PMC11802850

[6] Gibelalde A Amenabar Alonso A Pinar-Sueiro S Bilbao-Garay I Juaristi Eizmendi L Sampedro A . Albuminuria as a biomarker of severity in diabetic retinopathy and in the response to intravitreal treatment in diabetic macular edema. Int Ophthalmol. (2023) 43:2049–56. doi: 10.1007/s10792-022-02604-y. PMID: 36512296

[7] Zheng C Cao D Ma R Hao X Jin L Hu Y . Current status of multi-target therapeutics for diabetic macular oedema: mechanisms and outcomes from clinical trials. Diabetes Obes Metab. (2026) 28(3):1649–1660. doi: 10.1111/dom.70405, PMID: 41431856

[8] Zooravar D Shojaei S Mousavi A Soltani P Amiri BS Radkhah H . Novel lipid biomarkers and microvascular complications in patients with diabetes mellitus: a systematic review and meta-analysis. Clin Med Insights Endocrinol Diabetes. (2025) 18:11795514251365301. doi: 10.1177/11795514251365301. PMID: 40827201 PMC12357997

[9] He Y Zhou J Wang J Huang S Li H Cao J . Novel visceral obesity indicators and associated metabolic fingerprint in incident diabetic retinopathy. Invest Ophthalmol Visual Sci. (2025) 66:17. doi: 10.1167/iovs.66.12.17. PMID: 40919861 PMC12422393

[10] Chen J Li YT Niu Z He Z Xie YJ Hernandez J . Association of visceral obesity indices with incident diabetic retinopathy in patients with diabetes: prospective cohort study. JMIR Public Health Surveillance. (2024) 10:e48120. doi: 10.2196/48120. PMID: 38319705 PMC10879974

[11] Catak M Konuk SG Hepsen S . The cholesterol-HDL-glucose (CHG) index and traditional adiposity markers in predicting diabetic retinopathy and nephropathy. J Diabetes Invest. (2025) 16:1487–94. doi: 10.1111/jdi.70086, PMID: 40434226 PMC12315246

[12] Ergin E Dascalu AM Stana D Tribus LC Arsene AL Nedea MI . Predictive role of complete blood count-derived inflammation indices and optical coherence tomography biomarkers for early response to intravitreal anti-VEGF in diabetic macular edema. Biomedicines. (2025) 13(6). doi: 10.3390/biomedicines13061308. PMID: 40564027 PMC12189659

[13] Wasfy TE Soliman SS Hawash N Gad EA . Abdominal obesity as a predictor for response to anti-vascular endothelial growth factor therapy in patients with diabetic macular edema. (2025) 118(1):7–12. doi: 10.4103/ejos.ejos_25_24

[14] Sun JK Beaulieu WT Melia M Ferris FL Maturi RK Nielsen JS . Defining "strong" versus "weak" response to antivascular endothelial growth factor treatment for center-involved diabetic macular edema. Retina (Philadelphia Pa). (2023) 43:616–23. doi: 10.1097/IAE.0000000000003730. PMID: 36728692 PMC11040570

[15] Sun Q Zhao M Wang X Wang J Yang Y Liu L . The predictive value of insulin resistance surrogates for diabetic kidney disease in type 2 diabetes mellitus. Diabetes Ther. (2025) 16:1649–63. doi: 10.1007/s13300-025-01765-0. PMID: 40517339 PMC12317950

[16] Rył A Szylińska A Bohatyrewicz A Jurewicz A Pilarczyk B Tomza-Marciniak A . Relationships between indicators of metabolic disorders and selected concentrations of bioelements and lead in serum and bone tissue in aging men. Diabetes Metab Syndrome Obesity: Targets Ther. (2022) 15:3901–11. doi: 10.2147/DMSO.S387444. PMID: 36540347 PMC9759988

[17] Pan L Xu Q Liu J Gao Y Li J Peng H . Dose-response relationship between Chinese visceral adiposity index and type 2 diabetes mellitus among middle-aged and elderly Chinese. Front Endocrinol. (2022) 13:959860. doi: 10.3389/fendo.2022.959860. PMID: 36277708 PMC9579311

[18] Miaomiao S Journal CZJXM . Prospective association between visceral fat index and stroke risk in middle-aged and elderly people in China. (2025) 16(4):922–929.

[19] Jakubiak GK Badicu G Surma S Waluga-Kozlowska E Chwalba A Pawlas N . The visceral adiposity index and its usefulness in the prediction of cardiometabolic disorders. Nutrients. (2025) 17(14). doi: 10.3390/nu17142374. PMID: 40732999 PMC12298961

[20] Yu Y Tan T Yang W Xu Z Liu Y . Decoding the nonlinear association between visceral adiposity index and all-cause mortality: the mediating role of white blood cells and neutrophils. Int J Endocrinol. (2025) 2025:3116986. doi: 10.1155/ije/3116986. PMID: 41479855 PMC12753583

[21] Fonseka S Subhani B Wijeyaratne CN Gawarammana IB Kalupahana NS Ratnatunga N . Association between visceral adiposity index, hirsutism and cardiometabolic risk factors in women with polycystic ovarian syndrome: a cross-sectional study. Ceylon Med J. (2019) 64:111–7. doi: 10.4038/cmj.v64i3.8958. PMID: 32120461

[22] Choi KE Joung C Pahk KJ Kim H Pahk K . Metabolic activity of visceral adipose tissue is associated with age-related macular degeneration: a pilot (18)F-FDG PET/CT study. Front Endocrinol. (2023) 14:1322326. doi: 10.3389/fendo.2023.1322326, PMID: 38260144 PMC10801050

[23] Choi KE Joung C Yoon E Chung HW Pahk KJ Pahk K . Association between metabolic activity of visceral adipose tissue and retinal vein occlusion: a preliminary (18)F-FDG PET/CT study. Front Endocrinol. (2025) 16:1679216. doi: 10.3389/fendo.2025.1679216. PMID: 41113704 PMC12527876

[24] Das UN . Diabetic macular edema, retinopathy and age-related macular degeneration as inflammatory conditions. Arch Med Science: AMS. (2016) 12:1142–57. doi: 10.5114/aoms.2016.61918. PMID: 27695506 PMC5016593

[25] Savage SR McCollum GW Yang R Penn JS . RNA-seq identifies a role for the PPARβ/δ inverse agonist GSK0660 in the regulation of TNFα-induced cytokine signaling in retinal endothelial cells. Mol Vision. (2015) 21:568–76. PMC4443583 26015769

[26] Vinores SA Xiao WH Shen J Campochiaro PA . TNF-alpha is critical for ischemia-induced leukostasis, but not retinal neovascularization nor VEGF-induced leakage. J Neuroimmunology. (2007) 182:73–9. doi: 10.1016/j.jneuroim.2006.09.015. PMID: 17107717 PMC1800833

[27] Sun K Wernstedt Asterholm I Kusminski CM Bueno AC Wang ZV Pollard JW . Dichotomous effects of VEGF-A on adipose tissue dysfunction. PNAS. (2012) 109:5874–9. doi: 10.1073/pnas.1200447109. PMID: 22451920 PMC3326476

[28] Burlina P Paul W Liu TYA Bressler NM . Detecting anomalies in retinal diseases using generative, discriminative, and self-supervised deep learning. JAMA Ophthalmol. (2022) 140:185–9. doi: 10.1001/jamaophthalmol.2021.5557. PMID: 34967890 PMC8719271

[29] Busch C Katzmann JL Jochmann C Unterlauft JD Vollhardt D Wiedemann P . General health of patients with diabetic macular edema-the LIPSIA study. PloS One. (2021) 16:e0252321. doi: 10.1371/journal.pone.0252321. PMID: 34115786 PMC8195383

[30] Zhang M Wu J Wang Y Wu J Hu W Jia H . Associations between blood pressure levels and diabetic retinopathy in patients with diabetes mellitus: a population-based study. Heliyon. (2023) 9:e16830. doi: 10.1016/j.heliyon.2023.e16830. PMID: 37484372 PMC10360950

[31] Malerbi FK Mendes G Barboza N Morales PH Montargil R Andrade RE . Diabetic macular edema screened by handheld smartphone-based retinal camera and artificial intelligence. J Med Syst. (2021) 46:8. doi: 10.1007/s10916-021-01795-8. PMID: 34893931 PMC8664675

