# Supplementary material for: Systemic metabolic dysfunction is associated with local treatment failure: the role of visceral adiposity index in anti-VEGF resistance in diabetic macular edema
Source: Front Endocrinol (Lausanne). 2026 Mar 26;17:1801978. doi: 10.3389/fendo.2026.1801978 (PMC13061709; doi:10.3389/fendo.2026.1801978)
Supplement: Supplementary file 1 [file Table1.docx]

**Supplementary Table S1. Variance Inflation Factor (VIF) for Fully Adjusted Model (Model 3)**

| **Variable** | **VIF_Value** |
| --- | --- |
| **Visceral Adiposity Index** | **1.332** |
| **Age** | **1.147** |
| **Male Sex** | **1.188** |
| **Diabetes Duration** | **1.273** |
| **HbA1c** | **1.192** |
| **Baseline CRT** | **1.176** |
| **Baseline BCVA (logMAR)** | **1.142** |
| **Hypertension** | **1.031** |

**Note: All VIF values are < 5, confirming the absence of significant multicollinearity among the variables included in the multivariable logistic regression analysis. Individual components of VAI were excluded.**
