# Supplementary material for: Systemic metabolic dysfunction is associated with local treatment failure: the role of visceral adiposity index in anti-VEGF resistance in diabetic macular edema
Source: Front Endocrinol (Lausanne). 2026 Mar 26;17:1801978. doi: 10.3389/fendo.2026.1801978 (PMC13061709; doi:10.3389/fendo.2026.1801978)
Supplement: Supplementary file 2 [file Table2.docx]

**Supplementary Table S2. Hosmer-Lemeshow Goodness-of-Fit Test for Model Calibration**

| **Test** | **Model** | **Chi_Square** | **df** | **P_Value** | **Interpretation** |
| --- | --- | --- | --- | --- | --- |
| **Hosmer-Lemeshow Goodness-of-Fit** | **Fully Adjusted Model (Model 3)** | **12.334** | **8** | **0.137** | **Adequate Calibration (Fit)** |

**Note: The Hosmer-Lemeshow test evaluates how well the model's predicted probabilities match the observed outcomes. A p-value > 0.05 indicates no significant difference between predicted and observed values, suggesting adequate model calibration (goodness-of-fit).**
