# Supplementary material for: Systemic metabolic dysfunction is associated with local treatment failure: the role of visceral adiposity index in anti-VEGF resistance in diabetic macular edema
Source: Front Endocrinol (Lausanne). 2026 Mar 26;17:1801978. doi: 10.3389/fendo.2026.1801978 (PMC13061709; doi:10.3389/fendo.2026.1801978)
Supplement: Supplementary file 3 [file Table3.docx]

**Supplementary Table S3. Association Between VAI Quartiles and Treatment Response (Fully Adjusted Model)**

| **VAI Quartiles** | **Adjusted OR (95% CI) for Treatment Response** |
| --- | --- |
| **Q1 (Lowest Quartile)** | **1.00 (Reference)** |
| **Q2** | **0.42 (0.18 - 0.94)** * |
| **Q3** | **0.28 (0.12 - 0.65)** * |
| **Q4 (Highest Quartile)** | **0.08 (0.03 - 0.22)** * |

**Note: Indicates statistical significance at the 0.05 level. The model is fully adjusted for age, sex, diabetes duration, HbA1c, baseline CRT, baseline BCVA, and hypertension. OR = Odds Ratio; CI = Confidence Interval. Patients in the highest quartile (Q4) showed significantly reduced odds of positive treatment response compared to those in the lowest quartile (Q1).*
