# Supplementary material for: Systemic metabolic dysfunction is associated with local treatment failure: the role of visceral adiposity index in anti-VEGF resistance in diabetic macular edema
Source: Front Endocrinol (Lausanne). 2026 Mar 26;17:1801978. doi: 10.3389/fendo.2026.1801978 (PMC13061709; doi:10.3389/fendo.2026.1801978)
Supplement: Supplementary file 4 [file Table4.docx]

**Supplementary Table S4. Sensitivity Analysis Comparing Standardized VAI and CVAI for Identifying Poor Response**

| **Index** | **Adjusted OR (95% CI) per 1 SD Increase** | **AUC (95% CI) for Identifying Poor Response** |
| --- | --- | --- |
| **VAI (per 1 SD increase)** | **0.40 (0.26 - 0.57)** | 0.73 (0.67 - 0.79) |
| **CVAI (per 1 SD increase)** | **0.44 (0.30 - 0.63)** | 0.69 (0.63 - 0.75) |

**Note: Both VAI and CVAI were converted to Z-scores to calculate the Adjusted Odds Ratio per 1 Standard Deviation (SD) increase. OR = Odds Ratio; AUC = Area Under the Curve; VAI = Visceral Adiposity Index; CVAI = Chinese Visceral Adiposity Index. The model is adjusted for age, sex, diabetes duration, HbA1c, baseline CRT, baseline BCVA, and hypertension.**
